# Correction: Wang et al. Molecular Dissection of *TaLTP1* Promoter Reveals Functional *Cis*-Elements Regulating Epidermis-Specific Expression. *Int. J. Mol. Sci.* 2020, *21*, 2261

**DOI:** 10.3390/ijms262010215

**Published:** 2025-10-21

**Authors:** Guiping Wang, Guanghui Yu, Yongchao Hao, Xinxin Cheng, Jinxiao Zhao, Silong Sun, Hongwei Wang

**Affiliations:** State Key Laboratory of Crop Biology, Shandong Key Laboratory of Crop Biology, College of Agronomy, Shandong Agricultural University, Tai’an 271018, China; wgp03165@163.com (G.W.); yuguanghui2009@126.com (G.Y.); hychao96@126.com (Y.H.); 17863800651@163.com (J.Z.)

In the original publication [1], there was a mistake in Figure 1C as published. The representative photos did not precisely correspond to the panel labels of related promoter constructs. The corrected Figure 1 appears below. The authors state that the scientific conclusions are unaffected. This correction was approved by the Academic Editor. The original publication has also been updated.

## Figures and Tables

**Figure 1:**
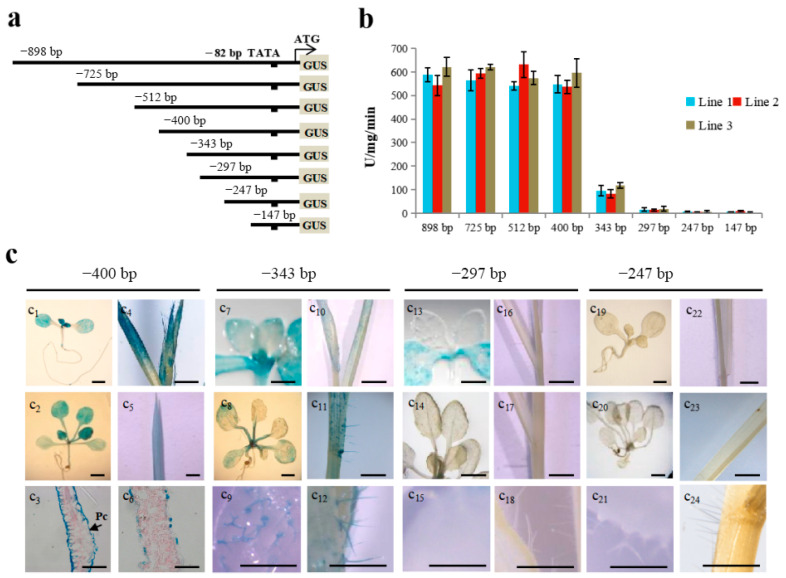
Activities of a *TaLTP1* promoter deletion series in transgenic *A. thaliana* and *B. distachyon*. (**a**) Each of the 5′ upstream fragments from the *TaLTP1* promoter was fused to the translational start site of the beta-glucuronidase reporter gene. (**b**) Quantitative GUS activity of two-week-old T_3_ transgenic *A. thaliana* seedlings with *TaLTP1* promoter deletions. 4-MUG was used as substrate in the assay. Three replicates were used for each transgenic line; each replicate contained at least 5 seedlings. (**c**) GUS activity of *A. thaliana* transformed with pBI121 vector harboring -400*TaLTP1::uidA* (c_1_–c_3_), -343*TaLTP1::uidA* (c_7_–c_9_), -297*TaLTP1::uidA* (c_13_–c_15_), -247*TaLTP1::uidA* (c_19_–c_21_); GUS activity of *B. distachyon* transformed with pCAMBIA 1391Z vector harboring -400*TaLTP1::uidA* (c_4_–c_6_), -343*TaLTP1::uidA* (c_10_–c_12_), -297*TaLTP1::uidA* (c_16_–c_18_), -247*TaLTP1::uidA* (c_22_–c_24_). The first roll represents GUS staining in young seedling leaves, the second roll represents GUS staining in adult leaves of 2- to 3-week-old seedlings, and the third roll represents expression in leaf pavement cells and trichome cells in true leaves. Scale bars are about 40 μm for C_3_ and C_6_, and about 1 mm for all others.

## References

[1] Wang G., Yu G., Hao Y., Cheng X., Zhao J., Sun S., Wang H. (2020). Molecular Dissection of *TaLTP1* Promoter Reveals Functional *Cis*-Elements Regulating Epidermis-Specific Expression. Int. J. Mol. Sci..

